# Natural Products and Biological Activity from Actinomycetes Associated with Marine Algae

**DOI:** 10.3390/molecules28135138

**Published:** 2023-06-30

**Authors:** Zijun Xiong, Rong Wang, Tengfei Xia, Shiqing Zhang, Shuai Ma, Zhikai Guo

**Affiliations:** 1 Hainan Key Laboratory of Tropical Microbe Resources, Institute of Tropical Bioscience and Biotechnology, Chinese Academy of Tropical Agricultural Sciences & Key Laboratory for Biology and Genetic Resources of Tropical Crops of Hainan Province, Hainan Institute for Tropical Agricultural Resources, Haikou 571101, China; xiongzijun@itbb.org.cn (Z.X.);; 2 Hainan Provincial Key Laboratory of Tropical Maricultural Technologies, Hainan Academy of Ocean and Fisheries Sciences, Haikou 571126, China; 3 Institute of Tropical Horticulture Research, Hainan Academy of Agricultural Sciences, Haikou 571100, China

**Keywords:** actinomycetes, marine algae, secondary metabolite, bioactive compound

## Abstract

Marine natural products have been recognized as the most promising source of bioactive substances for drug discovery research. This review illustrates the diversity of culturable actinobacteria associated with marine algae, their bioactivity and metabolites, and approaches to their isolation and determination of their biological properties. Furthermore, actinobacteria associated with marine algae are presented as a new subject for an extensive investigation to find novel and active natural products, which make them a potentially rich and innovative source for new drug development deserving more attention and exploration.

## 1. Introduction

More than 50% of new drug discoveries are derived from natural products and their derivatives, and natural products play an important role in drug discovery [1]. Over recent decades, natural marine products have been a fruitful field for organic chemistry research; more than 39,845 publications and 40,218 compounds have been deposited in the database of marine natural products research (http://pubs.rsc.org/marinlit, accessed on 5 June 2023). Marine organisms are excellent producers of secondary metabolites with diverse structures and bioactivities due to their special habitations and unique ecological conditions, such as low or high temperatures, low pH, high pressures, and high salt concentrations [2,3]. Marine microorganisms have taken the limelight as a producer of active natural products, including anti-bacterial, anti-fungal, anti-viral, enzyme, anti-biofilm, anti-cancer, anti-oxidant, and anti-inflammation substances [4]. Due to co-existing with their host and the possible production of compounds with novel structures and diverse activities, marine microorganisms associated with marine animals and plants have attracted much attention [5,6].

Marine algae have attracted attention as a source of new bioactive molecules of biomedical interest, and they provide suitable living conditions and abundant nutrition for different microorganisms, while in return, microorganisms may provide protection and ultimately survival value to their hosts by producing bioactive molecules, or by affecting the growth and evolution process via the signal transduction pathway [5,6,7,8,9]. Although algae-associated microorganisms have great potential for secondary metabolite synthesis, some related studies deserve more attention [10,11]. At present, the research on algae-associated microorganisms focuses on macroalgae-associated fungi, and more than 400 new natural products have been obtained from them, which have anti-tumor, anti-bacterial, anti-oxidant, and insecticidal activities, providing a rich and innovative source for new drug candidates [12,13]. However, studies on algae-associated actinomyces have been neglected [11].

Although some scholars have found a low abundance of actinomyces in culture-free analyses on the diversity of algal-associated bacteria [11,14,15], there are some reports on the diversity of algal-associated actinomyces and more reports on secondary metabolites from algal-associated actinomyces, most of which have novel structures and good biological activities. In this review, the diversity of culturable actinobacteria associated with marine algae and their metabolites are illustrated, as well as approaches to their isolation and the determination of their biological properties. Furthermore, the data analysis summarized in this article suggests that these actinomycetes associated with marine algae deserve more attention in terms of resource exploration and utilization.

## 2. Marine Actinomyces

When streptomycin, which had a significant effect on tuberculosis, was obtained from *Streptomyces* in the 1850s, more researchers focused on the resources and metabolites of actinobacteria. Actinobacteria, also called actinomycetes, are Gram-positive bacteria belonging to the phylum Actinobacteria, characterized by a mycelial morphology with branched hyphae and the ability to form spores [16]. However, some non-spore rare actinomycetes, such as *Salinisporas* and *Arthrobacter*, were also found. Actinomycetes are rich in soil, and a lot of research has been done in studying their secondary metabolites. They are famous for their ability to produce abundant metabolites, especially antibiotics [17,18,19]. As a major producer of antibiotics, actinomyces, especially *Streptomyces*, are still an area of interest in the search for new structures and active substances. 

Marine actinomyces were first proposed by Okami in 1986. The normal growth of marine actinomyces needs seawater, because they are isolated from the marine environment. Some marine actinomyces can grow in the laboratory without relying on seawater [20]. The distribution of marine actinomyces is very wide and they can be distributed in the deep sea, in shallow water, near the shore, and in the intertidal zone. Marine actinomyces have also been isolated from marine organisms, cold spring areas, mining areas, and deep sediments [21]. 

About half of the marine actinomycetes that produce active compounds have been reportedly isolated from marine sediments, the co-epiphytic sources of mangroves, and marine organisms [16]. The Ribosomal Database Project (RDP) classification contains 136 genera of marine actinomyces associated with marine organisms, identified in 2014 by 16S rRNA sequencing [22]. More than 536 secondary metabolites with anti-bacterial and cytotoxic properties have been isolated from 22 genera of marine actinomyces associated with marine organisms, and their structural types are mainly alkaloids, polyketides, and polypeptides [23]. The co-epiphytic hosts of these actinomyces are mainly invertebrates such as sponges, ascidians, and corals, with fewer originating from marine plants [23].

## 3. Actinomycetes Isolation from Marine Algae and Preliminary Identification

Algae are one of the major contributors to marine ecosystems, and are found in almost all parts of the coastal regions around the globe [24]. According to size, marine algae, also known as seaweed, can be divided into macroalgae and microalgae. Furthermore, macroalgae can be classified into brown algae (phaeophyceae), red algae (rhodophyceae), and green algae (chlorophyceae), due to their different pigmentations [25]. Additionally, algae can provide a relatively stable and nutrient-rich habitat for microorganisms that live on their surface, and harbor diverse groups of bacteria, depending on the species and season [26,27]. 

To obtain actinomycetes strains associated with marine algae, fresh algal samples should be placed in individual sterile plastic bags and transported at 4 °C; these samples should then be processed immediately. Firstly, fresh samples should generally be rinsed at least three times with sterile seawater and undergo surface sterilization with 70% EtOH for a few seconds, before being aseptically cut into small pieces and homogenized with a sterile pestle in moderate sterile seawater [15]. Secondly, the polished samples should be serially diluted and plated onto the isolation media. These prepared samples may be heat-treated (such as at 55 °C for 5 min) and selective culture media that contain antibiotics to inhibit the growth of Gram-negative bacteria and fungi may be chosen [28,29,30,31]. Thirdly, along with the prepared plates incubated at 28 °C for 2–8 weeks, the emergence of actinomycetes colonies should be assessed every week [15,28]. Then, colonies are selected, and pure cultures are obtained by repeated streaking on agar plates. Finally, actinomycetes-like strains are selected based on the colony morphology: solid density of colonies, growth inside the agar media, and steady border of the colonies [32].

To identify the isolated actinomycetes, the 16S rRNA gene sequencing method should be employed. In detail, the 16S rRNA gene of these isolated actinomycetes should be amplified by PCR with the universal primers 27F and 1492R, using their genomic DNA as templates. Then, these PCR products should be sequenced and submitted to blast the NCBI GenBank or the EMBL database using Basic Local Alignment Search Tool (BLAST) [28,31,32,33]. Lastly, these 16S rRNA sequences should be aligned and subjected to a phylogenetic analysis using MEGA software (version 11) [28].

## 4. Abundance of Actinomycetes Associated with Marine Algae

Marine algae harbor a diverse group of bacteria, depending on the season, species, and thallus structure [26,27], and the actinomycetes associated with marine algae are less studied. Ulfah et al. reported that a total of 15 actinobacteria were isolated from the red algae *Gelidiella acerosa* collected from Drini Gunungkidul Yogyakarta [34]. Rajivgandhi et al. reported that 50 endophytic actinomycetes were isolated from green algae *Cauler pataxifolia* [35] and 100 actinomycetes strains were isolated from brown macroalgae *Turbinaria ornata* and *Sargassum wightii*, collected from the southeast coast of Tamil Nadu, India [36]. Four actinomycetes strains associated with the brown algae *Sargassum cinereum* and three actinomycetes strains associated with the green algae *Codium dwarkense* were obtained by Majithiya et al. in 2022 [37]. Ninety actinomycetes strains were isolated from the brown algae *Laminaria ochroleuca* by Girão et al. [29]. Thirty-six actinomycetes were obtained from the marine brown algae *Laminaria saccharina* by Wiese et al. from the Baltic Sea, Germany [15].

According to the statistics from the literature, 22 genera (*Aeromicrobium, Agrococcus*, *Amycolatopsis*, *Arthrobacter*, *Brachybacterium*, *Citricoccus*, *Isoptericola*, *Kocuria*, *Labedella*, *Leifsonia*, *Microbacterium*, *Microbispora*, *Micrococcus*, *Micromonospora*, *Nocardiopsis*, *Nonomuraea*, *Phycicola*, *Rhodococcus*, *Salinibacterium*, *Salinispora*, *Sanguibacter*, and *Streptomyces*) in 11 families (*Dermabacteraceae*, *Jonesiaceae*, *Microbacteriaceae*, *Micrococcaceae*, *Micromonosporaceae*, *Nocardiaceae*, *Nocardiopsaceae*, *Promicromonosporaceae*, *Pseudonocardiaceae*, *Streptomycetaceae* and *Streptosporangiaceae*) of cultivable actinomycetes have been obtained from marine algae (Table 1). Among them, *Aeromicrobium tamlense* [38], *Amycolatopsis antarctica* [39], *Agrococcus jejuensis* [40], *Labedella gwakjiensis* [41] and *Phycicola gilvus* [42] are the most recent species of actinomycetes to be isolated from marine algae. The genus *Streptomyces* is widespread, and dominant strains related to marine algae and the actinomycetes associated with brown alga are relatively richer than those associated with green alga or red alga [15,29,43]. It was reported that more than 60 different genera of pure cultured marine actinomyces associated with sponges or corals have been obtained, respectively [21,44]. Although the abundance of actinomycetes associated with algae is less than that of those associated with sponges or corals, marine algae are a good source for isolating novel and rare actinobacteria deserving more attention and investment.

## 5. Biological Activities of the Actinomycetes Associated with Marine Algae

The most studied biological activity of actinomycetes associated with marine algae is anti-bacterial activity. As the report from Wiese et al. in 2009 showed [15], 36 actinobacteria, obtained from the marine brown algae *Laminaria saccharina,* showed different inhibition capacities of *Bacillus subtilis*, *Escherichia coli*, *Staphylococcus lentus* and/or *Candida albicans*. Of 100 actinomycetes, 40 isolated from brown macroalgae *Turbinaria ornata* and *Sargassum wightii* were active in antagonistic activity against various clinical pathogens [36]. Of a total of 15 actinobacteria, isolated from the red algae *Gelidiella acerosa*, 8 showed inhibition against *Vibrio alginolyticus* [34]. Of 50 endophytic actinomycetes, 20 isolates isolated from green algae *Cauler pataxifolia* showed antimicrobial activity against urinary tract infections bacteria (including *E. coli, Proteus mirabilis, Pseudomonas aeruginosa, Kilebsiella pneumonia,* and *Enterobacter* sp.) and the strain DMS 3 showed the best anti-bacterial activity among them [35]. Girão et al. (2019) also obtained 90 actinobacterial strains from brown algae *Laminaria ochroleuca*; 45 isolates inhibited the growth of *C. albicans* and/or *Staphylococcus aureus,* and 28 extracts among them affected the viability of at least one human cancer cell line (breast carcinoma T-47D or neuroblastoma SH-SY5Y) and non-carcinogenic endothelial cell line (hCMEC/D3) [29]. The crude extract and partially purified compounds from *Nocardiopsis* sp. DMS 2 were shown to have high inhibition activities against biofilm-forming *K. pneumoniae* [45].

Some actinomycetes associated with marine algae have been reported to show special enzyme activities, flocculating activity, and heavy metal sorption. *Streptomyces* sp. SNAJSM6 not only produced 56 U/mL of α-amylase, but also showed excellent anti-bacterial activity against selected pathogenic bacteria (*P. aeruginosa*, *Enterobacter* sp., *Salmonella* sp., and *Micrococcus luteus*) [46]. *Nocardiopsis* sp. GRG 3 showed a maximum flocculating activity of 80.90% with glucose, and the yield was 4.52 g/L. Furthermore, its heavy metal sorption effectively removed 55.90% Cd, 85.90% Cr, 74.7% Pb, and 51.90% Hg [47]. *Micrococcus* sp. GNUM-08124 could use agar as the sole carbon source, and showed higher agarase activity when cultured in an oligotrophic culture medium than in a rich media [48]. *Streptomyces* sp. SNJASM6 not only showed significant emulsification activities with tween 20, coconut oil, and xylene (which are the subsequent substrates of surfactant, oils, and hydrocarbons respectively), but also showed activity against bacterial pathogens including *E. coli*, *Bacillus cereus*, *P. aeruginosa*, *Klebsiella pneumoniae* and *C. albicans* [49].

As summarized in Table 2, 27 bioactive strains from the actinomycetes associated with marine algae exhibit diverse biological activities, such as anti-fungal, anti-bacterial, anti-inflammatory, anti-tuberculosis, cytotoxicity, and herbicidal activity. Most of the bioactive strains were *Streptomyces* spp., and they were mainly isolated from brown algae and green algae.

## 6. Bioactive Metabolites from Actinomycetes Associated with Marine Algae

There are 82 compounds that have been isolated from 20 actinomycetes associated with marine algae. Additionally, 35 new metabolites have also been isolated from these actinobacteria. Depending on their chemical structure, the metabolites are classified into polyketides, peptides, glycoglycerolipids, alkaloids, and pyrones. These compounds also showed diverse biological activities, and they are described below in the order of the Latin names of their producers.

### 6.1. Bioactive Metabolites from Streptomyces

**Figure 1:**
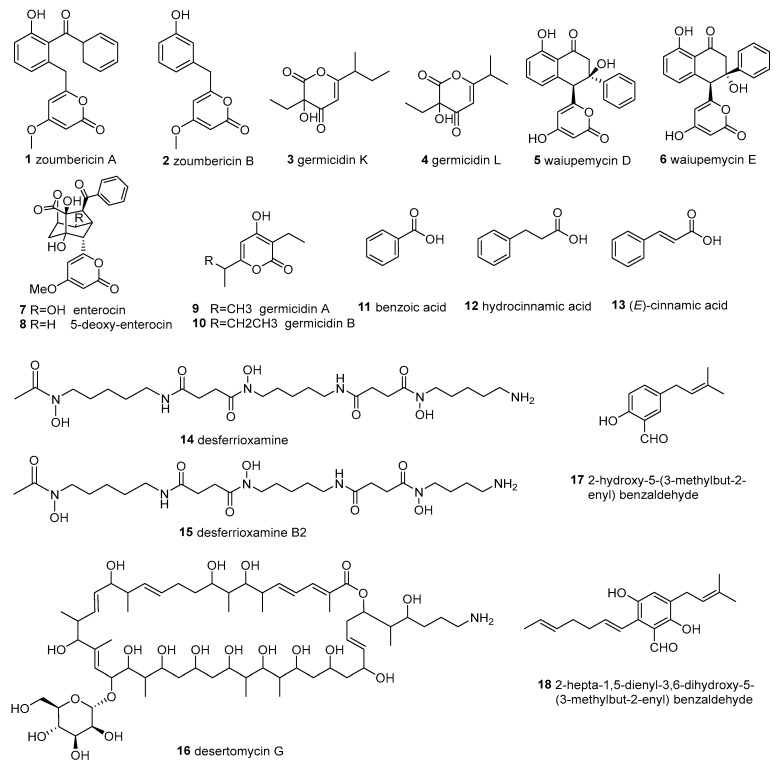
Structures of compounds **1**–**18**.

*Streptomyces ambofaciens* BI0048 was isolated from the red algae *Laurencia glandulifera*, collected in Zoumberi Bay, south of Nea Makri, Attiki, Greece. Four new α-pyrone polyketides (zoumbericin A (**1**) and B (**2**), germicidin K (**3**) and L (**4**)) along with wailupemycin D (**5**) and E (**6**), enterocin (**7**) (also named vulgamycin), 5-deoxy-enterocin (**8**), germicidin A (**9**) and B (**10**), benzoic acid (**11**), hydrocinnamic acid (**12**), and (*E*)-cinnamic acid (**13**) (Figure 1) have been isolated and identified from the organic extract of the strain’s fermentation broth [68]. It was reported that compound **7** showed herbicidal activity [69] and weak anti-bacterial activity against *M. luteus* [71]. Compound **8** was reported to be active against *Sarcina lutea*, *S. aureus*, *K. pneumoniae*, and *Vibrio percolans* [70]. Unfortunately, the ten compounds **1**–**10** were proven inactive in terms of their anti-bacterial activities against the epidemic methicillin-resistant strain EMRSA-15 and *E. coli,* and showed poor cytotoxic activities against human cancer cell lines MCF7 (breast adenocarcinoma) and A549 (lung carcinoma) [68].

As regards *Streptomyces albidoflavus* KC180, isolated from the marine brown algea *Carpodesmia tamariscifolia*, collected from the Atlantic coast of Morocco, the organic extracts of fermentation broths showed anti-bacterial activity to methicillin-resistant *Staphylococcus aureus* (MRSA), imipenem-resistant *Acinetobacter baumannii* and carbapenem-resistant *Pseudomonas aeruginosa*. Further research showed that it produced the active metabolite desferrioxamine B (**14**) and its new derivative desferrioxamine B2 (**15**) (Figure 1) against multidrug-resistant bacteria [51].

*Streptomyces althioticus* MSM3 was isolated from intertidal macroalgae brown algae *Ulva* sp. Collected from the Cantabrian Sea in Pedreña. A new compound desertomycin G (**16**) (Figure 1) was separated from its liquid fermentation with an R5A medium. Compound **16** exhibited inhibitory activities against clinical infection pathogens, including the strong inhibition of Gram-positive bacteria (*Corynebacterium urealyticum*, *S. aureus*, *Streptococcus pneumoniae*, *Streptococcus pyogenes*, *Enterococcus faecium*, *Enterococcus faecalis*, *Clostridium perfringens* and *Mycobacterium tuberculosis*) and the moderate inhibition of Gram-negative bacteria (*Bacteroides fragilis*, *Haemophilus influenzae* and *Neisseria meningitidis*). Additionally, it can decrease the viability of tumor cell lines MCF-7 (human breast adenocarcinoma) and DLD-1 (colon carcinoma) [67].

*Streptomyces atrovirens* PK288-21, obtained from the rhizosphere of the *Undaria pinnatifida,* was collected from the coast of Korea. 2-hydroxy-5-(3-methylbut-2-enyl) benzaldehyde (**17**, a new benzaldehyde derivative) and 2-hepta-1,5-dienyl-3,6-dihydroxy-5-(3-methylbut-2-enyl) benzaldehyde (**18**) (Figure 1) were obtained from the cultivation of the strain by Cho et al. in 2012. Both the compounds showed anti-bacterial activities against bacterial fish pathogens, including *Lactococcus garvieae*, *Streptococcus iniae*, *Streptococcus parauberis*, *Edwardsiella tarda*, *Vibrio harveyi* and *V. anguillarum*, with MIC values ranging from 20.0 to 128.0 μg/mL [58].

**Figure 2:**
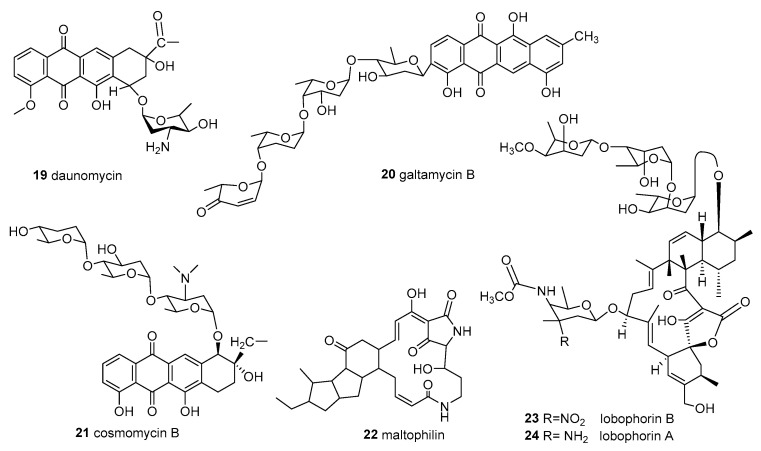
Structures of compounds **19**–**24**.

*Streptomyces carnosus* M-40 and *Streptomyces cyaneofuscatus* M-27 were isolated from brown macroalgae *Cystoseira baccata* and displayed strong antibiotic activities against Gram-positive and Gram-negative bacteria and fungi. *S. cyaneofuscatus* M-27 produced several antitumor antibiotics of the anthracycline family, of which daunomycin (**19**), galtamycin B (**20**), and cosmomycin B (**21**) (Figure 2) were identified. An anti-fungal macrolactam maltophilin (**22**) (Figure 2) was also identified from its ethyl acetate extracts. In addition, compounds **14**, **15**, and lobophorine B (**23**) (Figure 2) were separated from *S. carnosus* M-40 [43]. Interestingly, compounds **23** and lobophorine A (**24**) were first isolated from the unidentified actinomycete associated with brown algae [55].

**Figure 3:**
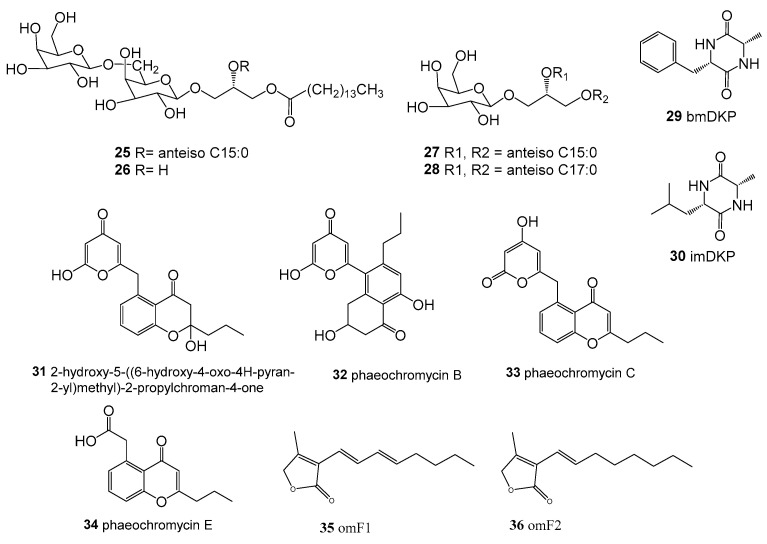
Structures of compounds **25**–**36**.

*Streptomyces coelescens* PK206-15 was isolated from the seaweed *Laminaria japonica* rhizosphere, collected from the coast of Korea. Its crude extracts showed anti-fouling activity against *Ulva pertusa* zoospore settlement with EC_50_ < 5 mg/mL. Four glycoglycerolipids (**25**–**28**) (Figure 3) were obtained from its crude extract, and they were active against the zoospores of *U. pertusa*, the mussel *Mytilus edulis*, the diatom *Navicula annexa,* and fouling bacteria, with an EC_50_ ranging from 0.005 to 0.2 µg/mL [54].

*Streptomyces praecox* 291-11 was isolated from seaweed brown algae *Undaria pinnatifida* rhizosphere, collected from a 10 m depth along the coast of Korea. The strain was screened out via its anti-fouling activity against the marine seaweed *U. pertusa* and fouling diatom *N. annexa*, and then two anti-fouling compounds, (6*S*,3*S*)-6-benzyl-3-methyl-2,5-diketopiperazine (**29**, bmDKP) and (6*S*,3*S*)-6-isobutyl-3-methyl-2,5-diketopiperazine (**30**, imDKP) (Figure 3), were isolated from its crude extract after optimization of the medium composition. In addition, the two compounds showed a therapeutic ratio (LC_50_/EC_50_) able to inhibit zoospores of 17.7 and 21, respectively. Furthermore, they showed a therapeutic ratio able to inhibit diatoms of 263 and 120.2, respectively [59].

*Streptomyces sundarbansensis* WR1L1S8 was associated with brown algae *Fucus* sp., collected along the Bejaia coastline, Algeria. A new polyketide (**31**) with three known phaeochromycins (**32**–**34**) (Figure 3) was obtained from agar solid fermentation. The new compound **31** was the major metabolite under culture conditions, and its activity against the pathogenic MRSA was prominent, with an MIC of 6 μΜ. In addition, the compounds **31**, **33**, and **34** also showed potent activity against *E. coli* and *P. aeruginosa* [53].

*Streptomyces violaceoruber* SCH-09 was isolated from brown algae *Undaria pinnatifida* (collected from the coast of Korea) and screened out for its anti-fouling activities from culture extracts. Two furanone derivatives, omF (**35**) and omF2 (**36**) (Figure 3), were obtained as active compounds from its culture extracts, and they showed anti-fouling activities against zoospores of *U. pertusa*, mussel *M. edulis,* and diatom *N. annexa*, with an EC_50_ range of 0.02–0.1 μM [60].

**Figure 4:**
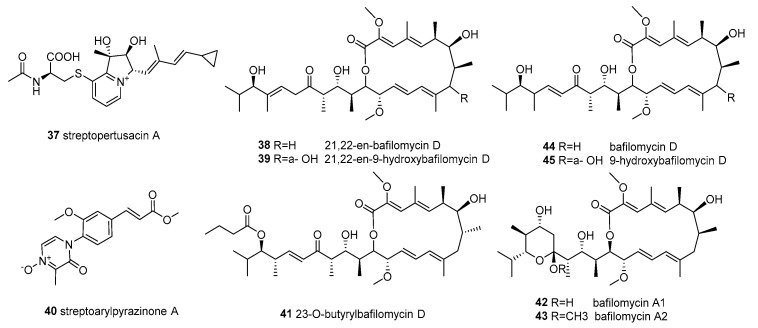
Structures of compounds **37**–**45**.

*Streptomyces* sp. HZP-2216E was obtained from sea lettuce *Ulva pertusa*, collected from the South China Sea close to Shanwei City (Guangdong, China). It produced different bioactive metabolites in different culture conditions (Gause’s liquid medium with sea salt liquid medium and glucose–yeast–malt solid medium). A unique indolizinium alkaloid streptopertusacin A (**37**) and four new compounds 21,22-en-bafilomycin D (**38**), 21,22-en-9-hydroxybafilomycin D (**39**), streptoarylpyrazinone A (**40**) and 23-*O*-butyrylbafilomycin D (**41**), together with the known bafilomycin A_1_ (**42**), bafilomycin A_2_ (**43**)_,_ bafilomycin D (**44**) and 9-hydroxybafilomycin D (**45**) (Figure 4) (in total 9 compounds), were separated from the fermentation extracts. In addition, all the compounds showed different activities against the growth of MRSA [65]. The new compounds **38** and **39** showed potent activity against the proliferation of glioma U251 and C6 cells, with IC_50_ 0.12–1.08 µM, and their MIC values against MRSA were 12.5 mg/mL. The four compounds **41**, **42**, **44**, and **45** showed potent activity in suppressing the proliferation of the four tested glioma cell lines with IC_50_ values of 0.35 to 2.95 µM [66].

**Figure 5:**
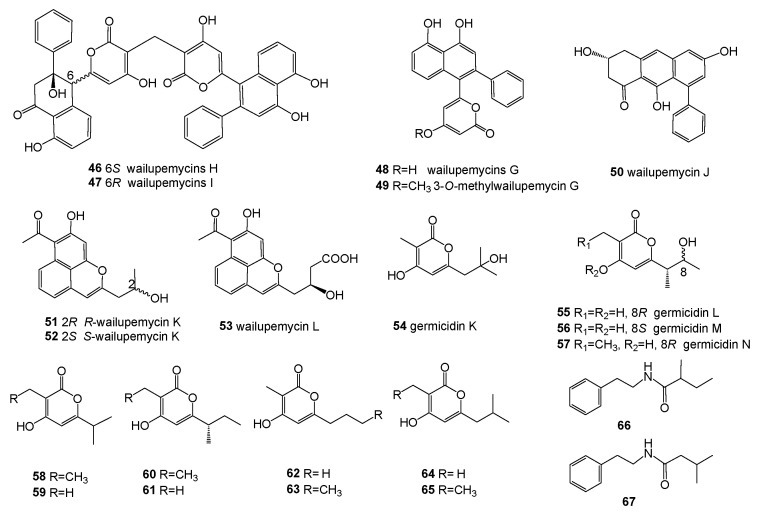
Structures of compounds **46**–**67**.

*Streptomyces* sp. OUCMDZ-3434 was isolated from the marine green algae *Enteromorpha prolifera*, collected from Zhanqiao Beach, Qingdao, Shandong Province, China. The EtOAc extract of its fermentation broth exhibited significant α-glucosidase inhibitory activity at 50 μg/mL. In addition, two new epimeric polyketides (wailupemycins H (**46**) and I (**47**)) with an unusual carbon skeleton, along with the three known compounds **5**, **6**, and wailupemycins G (**48**) (Figure 5), were obtained in the chemical study. Furthermore, the five compounds **5**, **6**, and **46**–**48** showd stronger inhibition of α-glucosidase and lower cytotoxicity than acarbose, with the IC_50_/CC_50_ values of 19.7/279.8, 8.3/1317.2, 988.7/2750.0, 392.5/2975.3, and 239.3/2953.8, respectively [61].

The continuous study of the remaining part of the EtOAc extract led to the isolation and identification of five new polyketides, 3-*O*-methylwailupemycin G (**49**), wailupemycin J (**50**), *R*-wailupemycin K (**51**), *S*-wailupemycin K (**52**) and wailupemycin L (**53**) (Figure 5), along with the known compounds **7** and **8**. In addition, compound **49** showed a-glucosidase inhibition with an IC_50_ 863.6 μM, and **52** was cytotoxic on the HeLa cell with an IC_50_ 8.2 mM. Furthermore, **8**, **51**, and **52** showed inhibitory activities against the H1N1 virus, with inhibition rates of 47.8%, 42.5%, and 60.6% at a concentration of 50 μM, respectively [62].

*Streptomyces* sp. OUCMDZ-3436 was isolated from the green algae *Enteromorpha prolifera*, which was collected from Zhanqiao Beach, Qingdao, Shandong Province, China. Four new α-pyrones (**54**–**57**) and eight known analogues (**58**–**65**) (Figure 5) were identified from its secondary metabolites, and compounds **54**–**65** showed no anti-bacterial activity against the 15 tested pathogenic organisms [63].

*Streptomyces* sp. PNM-9 isolated from the brown algae *Dictyota* sp. exhibited the ability to inhibit the in vitro growth of phytopathogens *Burkholderia glumae* and *Burkholderia gladioli*. Two known compounds (**66**, **67**) (Figure 5) were identified from the organic extract of a 15-day LB media culture, and were active against the rice pathogenic bacteria *B. glumae* with MICs of 2.43 mM and 1.21 mM, respectively [52].

**Figure 6:**
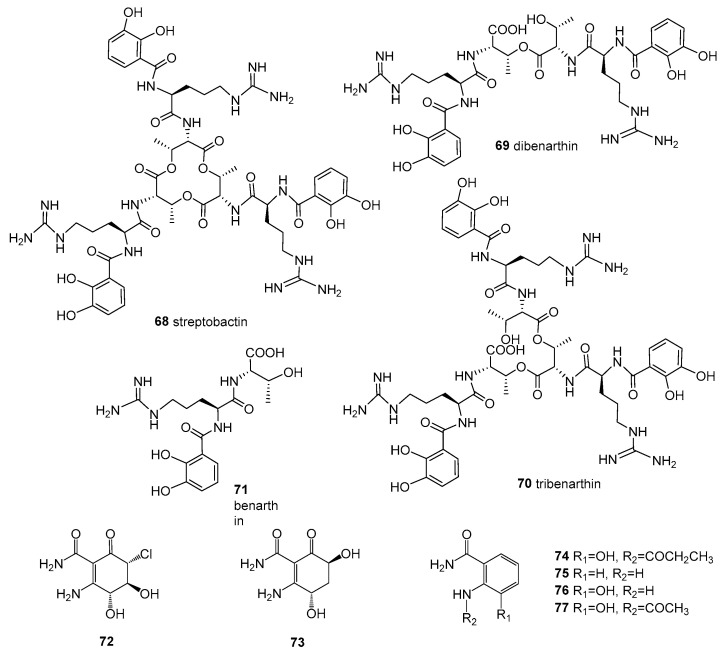
Structures of compounds **68**–**77**.

*Streptomyces* sp. YM5-799 was isolated from the surface of brown algae, collected from Hokkaido in north Japan. Three new catechol-type siderophores, streptobactin (**68**), dibenarthin (**69**), and tribenarthin (**70**), along with a known benarthin (**71**) (Figure 6), were obtained from the culture broth (ASG medium containing 0.1 μM FeCl_3_) of the strain. Compounds **68**, **69**, and **71** shoed an Fe-chelating activity, with the ED_50_ values 156, 117, and 937 μM, comparable to that of deferoxamine mesylate (ED_50_ = 195 μM) using a CAS assay [50].

*Streptomyces* sp. ZZ502 is associated with the green algae *Ulva conglobatea* growing on rocks on the coast of Zhoushan Archipelago in the East China Sea. Three new compounds, **72**–**74**, together with three known benzamide derivatives, **75**–**77** (Figure 6), were isolated from the solid culture extract. None of these isolated compounds showed activity in inhibiting the proliferation of glioma cells or the growth of MRSA, *E. coli*, or *C. albicans* [64].

### 6.2. Bioactive Metabolites from Non-Streptomyces

**Figure 7:**
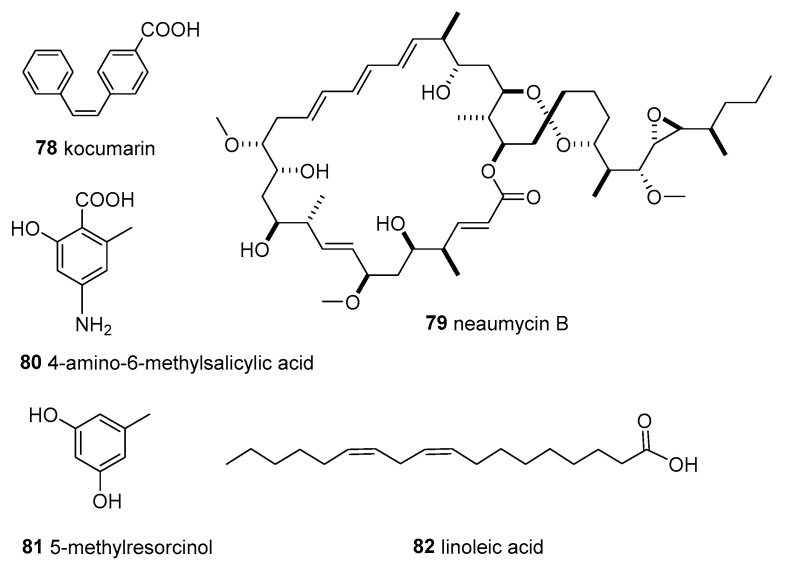
Structures of compounds **78**–**82**.

*Kocuria marina* CMG S2, associated with the brown seaweed *Pelvetia canaliculata* that grows on the rocks of Sonmiani Beach (Karachi, Pakistan), had remarkable antimicrobial activity. A new compound, 4-[(*Z*)-2 phenyl ethenyl] benzoic acid (**78**, kocumarin) (Figure 7), was isolated. Importantly, kocumarin demonstrated prominent and rapid growth inhibition against all tested fungi and pathogenic bacteria, including MRSA, with an MIC against fungi of 15–25 μg/mL and against bacteria of 10–15 μg/mL [56].

*Micromonospora* sp. CNY-010 was isolated from the surface of the brown algae *Stypopodium zonale*, collected from the Bahamas Islands. A new 28-membered macrolide containing 19 chiral centers named neomycin B (**79**) (Figure 7) was obtained from liquid fermentation. Compound **79** showed potent cytotoxicity, and was moderately active against RPMI-8226, a myeloma cell line involved in multiple myeloma [33].

*Nocardiopsis* sp. AS23C was isolated from brown algae *Sargassum arnaudianum*, collected in the Red Sea at the Hurghada coast, Egypt. The extract of this strain exhibited anti-bacterial activity against *B. subtilis, S. aureus,* and *Streptomyces viridochromogenes* Tü 57. Furthermore, a new phenolic acid derivative, 4-amino-6-methylsalicylic acid (**80**), and a new bacterial secondary metabolite, 5-methylresorcinol (**81**), along with linoleic acid (**82**) (Figure 7), were obtained from the crude extract [57].

The unidentified actinomycete CNC-837 was isolated from the surface inoculum of brown algae *Lobophora variegate,* which was collected from the Caribbean and produced two new macrolides. Two new compounds, **23** and **24** (Figure 2), showed anti-inflammatory activity, inhibiting topical PMA-induced edema in the mouse ear assay [55].

Natural products are a large resource for the development of drugs, and also a promising area for therapeutic agents [1,12,72]. Combining the microbial versatility and particularities of the marine environment, marine microorganisms have been considered to be the most promising natural source for drug discovery [13,72]. Marine actinomyces have shown an excellent biosynthetic ability to generate bioactive metabolites [23]. The most studied marine actinomyces are *Streptomyces*. Up to 2016, 547 new compounds had been isolated from marine *Streptomyces* [73]. Thereafter, more than 100 new compounds were added every year (except for 80 new compounds in 2020), and by 2021, more than 1196 new compounds had been obtained from marine *Streptomyces* [12,74,75,76,77]. These compounds included alkaloids, polyketides, halogens, terpenoids, and peptides, among which most compounds exhibited tumor cytotoxicity, anti-bacterial, anti-malarial and anti-parasitic activates, glycosidase inhibition, and other biological activities. Furthermore, *Nocardiopsis* was also an important source of secondary metabolites of marine actinomyces. According to the statistics, 67 natural products had been obtained from marine *Nocardiopsis* by 2019, with structures including pyranone, diketopiperazine, polypeptide, and so on [78]. The compounds summarised in this review were mainly derived from *Streptomyces*, followed by *Nocardiopsis* and *Micromonospora*, which is consistent with the study of marine actinomyces. In addition, the secondary metabolites of the first obligate marine actinomycete genus *Salinispora* have been found with 30 different structures, including Salinosporamide A [79], which was approved by the U.S. Food and Drug Administration (FDA) as an orphan drug for the treatment of multiple myeloma (Marizomib). *Salinispora* was mainly distributed in tropical and subtropical marine sedimentary environments, and was also found in marine sponges, sea squirts, and corals [80]. *Salinispora* recently proved to be abundant in Hainan Xisha marine algae, and may provide a rich and innovative source for new drug candidates [28].

Related to the source of actinomycetes associated with algae, it is clear that the abundance of actinomycetes that are associated with algae was less than that of those associated with sponges or corals [21,44]. However, 82 naturally occurring products, including 35 new ones, have been obtained from only 20 strains of the actinomycetes associated with marine algae. Many strains isolated from marine algae related to this review have not yet studied for their secondary metabolites, especially the seven remaining active strains summarized in Table 2. On the other hand, marine algae are broadly distributed in the ocean, with a great diversity of between 30,000 and more than 1 million different species [81]; in other words, there is much more scope for the resources of actinomycetes associated with marine algae to be studied. Furthermore, new actinomycetes resources and their biosynthetic potential are an untapped source of novel molecules and natural products. In conclusion, actinomycetes associated with marine algae are a good source for isolating novel and bioactive natural products deserving more attention and investment.

## 7. Conclusions

Marine algae have emerged as a vast source of bioactive metabolites and unique structures since marine resources have been paid attention to. The interesting ecological relationship between algae and associated microorganisms has since been addressed. In addition, the research on new natural products derived from algae-associated fungi is focused, and a large number of natural products with anti-tumor, anti-bacterial, anti-oxidant, and insecticidal activities have been obtained to provide a rich resource for new drug candidates. In this review, we summarized the abundance and bioactivity of actinomycetes associated with marine algae, and assessed the secondary metabolites for the chemistry and bioactivity of the natural products found in them. In total, 22 genera in 11 families of cultivable actinomycetes were obtained from marine algae, and they exhibit diverse biological activities, such as anti-bacterial activity, anti-fungal activity, anti-inflammatory, anti-tuberculosis, cytotoxicity, herbicidal activity, special enzyme activities, flocculating activity, and heavy metal sorption. From these actinomycetes, 82 naturally occurring products, including 35 new ones, have been obtained, and most of them show a variety of bioactivities. It is noteworthy that brown algae are the most representative samples from which actinomycetes are isolated, and *Streptomyces* spp. are the main producers of these metabolites so far. The actinomycetes associated with marine algae represent a new structure and a new source of bioactive natural products; however, they are still underexplored. Optimistically, future research on actinomycetes associated with marine algae may yield new developments and even more amazing breakthroughs.

## Figures and Tables

**Table 1:** List of actinobacterial genera associated with marine algae.

Isolation Source	Actinobacterial Genera	References
Brown algae	*Amycolatopsis*, *Arthrobacter*, *Isoptericola*, *Kocuria*, *Labedella*, *Leifsonia*, *Microbacterium*, *Microbispora*, *Micrococcus*, *Micromonospora*, *Nocardiopsis*, *Nonomuraea*, *Rhodococcus*, *Sanguibacter*, *Streptomyces.*	[15,29,30]
Red algae	*Brachybacterium*, *Citricoccus, Micrococcus*, *Salinibacterium*, *Streptomyces.*	[15]
Green algae	*Agrococcus*, *Arthrobacter*, *Brachybacterium*, *Micromonospora*, *Nocardiopsis*, *Rhodococcus*, *Salinibacterium*, *Salinispora*, *Streptomyces*.	[15,28]
Other algae	*Aeromicrobium*, *Agrococcus*, *Amycolatopsis*, *Labedella*, *Micromonospora*, *Nonomuraea*, *Phycicola*, *Rhodococcus*, *Salinispora*, *Streptomyces*.	[28,39,40,41,42]

**Table 2:** The bioactive strains from actinomycetes associated with marine algae.

Host	Location	Isolates	Biological Activity	References
**Brown algae**				
*Analipus japonicus* (Harvey) Wynne	Hokkaido, Japan	*Streptomyces* sp. YM5-799	Fe-chelating activity	[50]
*Carpodesmia tamariscifolia*	Atlantic coast of Morocco	*Streptomyces albidoflavus* KC180	anti-bacterial activity	[51]
*Cystoseira baccata*	Cantabrian Sea	*Streptomyces cyaneofuscatus* M-27	anti-bacterial activity; anti-fungal activity	[43]
		*Streptomyces carnosus* M-40	anti-bacterial activity; anti-fungal activity; anti-inflammatory; antituberculosis	
*Dictyota* sp.	Colombian Caribbean Sea	*Streptomyces* sp. PNM-9	anti-bacterial activity	[52]
*Fucus* sp.	Bejaia coastline, Algeria	*Streptomyces sundarbansensis* WR1L1S8	anti-bacterial activity	[53]
*Laminaria japonica*	Coast of Korea	*Streptomyces coelescens* PK206-15	antifouling activity	[54]
*Lobophora variegate*	Caribbean	unidentified actinomycete CNC-837	anti-inflammatory activity	[55]
*Pelvetia canaliculata*	Sonmiani Beach, Karachi, Pakistan	*Kocuria marina* CMG S2	anti-bacterial activity; anti-fungal activity	[56]
*Sargassum arnaudianum*	Red Sea at Hurghada coast, Egypt	*Nocardiopsis* sp. AS23C	anti-bacterial activity	[57]
*Sargassum myriocystum*	Tamil Nadu, Rameshwaram, India	*Streptomyces* sp. SNJASM6	anti-bacterial activity; α-amylase; emulsification activities with tween 20, coconut oil, and xylene	[46,49]
*Stypopodium zonale*	Bahamas Islands	*Micromonospora* sp. CNY-010	cytotoxicity	[33]
*Turbinaria ornata or Sargassum wightii*	Tamil Nadu, Rameshwaram, India	*Nocardiopsis* sp. GRG1	anti-bacterial activity	[36]
		*Nocardiopsis* sp. GRG2	anti-bacterial activity	[31]
		*Nocardiopsis* sp. GRG3	flocculating activity; heavy metal sorption	[47]
*Undaria pinnatifida*	Coast of Korea	*Streptomyces atrovirens* PK288-21	anti-bacterial activity	[58]
		*Streptomyces praecox* 291-11	antifouling activity	[59]
		*Streptomyces violaceoruber* SCH-09	antifouling activities	[60]
**Green algae**				
*Caulerpa racemosa*	Tamil Nadu, Rameshwaram, India	*Nocardiopsis* sp. DMS 2	anti-bacterial activity	[45]
*Cauler pataxifolia*	Tamil Nadu, Rameshwaram, India	unidentified actinomycete DMS 3	anti-bacterial activity	[35]
*Enteromorpha compressa*	East Sea of Korea	*Micrococcus* sp. GNUM-08124	agarase activity	[48]
*Enteromorpha prolifera*	Zhanqiao Beach, Shandong, China.	*Streptomyces* sp. OUCMDZ-3434	inhibitions of α-glucosidase; cytotoxicity; anti-viral activity	[61,62]
		*Streptomyces* sp. OUCMDZ-3436	anti-bacterial activity	[63]
*Ulva conglobatea*	East China Sea	*Streptomyces* sp. ZZ502	-	[64]
*Ulva pertusa*	South China Sea, Guangdong, China	*Streptomyces* sp. HZP-2216E	anti-bacterial activity; cytotoxicity	[65,66]
*Ulva* sp.	Cantabrian Sea, Pedreña	*Streptomyces althioticus* MSM3	anti-bacterial activity; cytotoxicity	[67]
**Red algae**				
*Laurencia glandulifera*	Zoumberi Bay, Attiki, Greece	*Streptomyces ambofaciens* BI0048	herbicidal activity; anti-bacterial activity	[68,69,70]

## Data Availability

All data have been included in this review.
